# Methyl 4-methyl-2-oxo-1,2,5,6-tetra­hydro-4*H*-pyrrolo[3,2,1-*ij*]quinoline-6-carboxyl­ate

**DOI:** 10.1107/S1600536809029948

**Published:** 2009-07-31

**Authors:** Yulia A. Zhuravleva, Anatolij V. Zimichev, Margarita N. Zemtsova, Victor B. Rybakov, Yurij N. Klimochkin

**Affiliations:** a Samara State Technical University, Molodogvardeyskay Str. 244, 443100 Samara, Russian Federation; b Department of Chemistry, Moscow State University, 119992 Moscow, Russian Federation

## Abstract

In the title mol­ecule, C_14_H_15_NO_3_, the six-membered heterocyclic ring exhibits an envelope conformation. In the crystal, C—H⋯π inter­actions link the mol­ecules into centrosymmetric dimers, and weak inter­molecular C—H⋯O hydrogen bonds link these dimers into columns propagated along [100].

## Related literature

For details of the synthesis, see: Zhuravleva *et al.* (2009 ▶). For a related structure, see: Bond *et al.* (1979 ▶). For a description of the Cambridge Structural Database, see: Allen (2002 ▶).
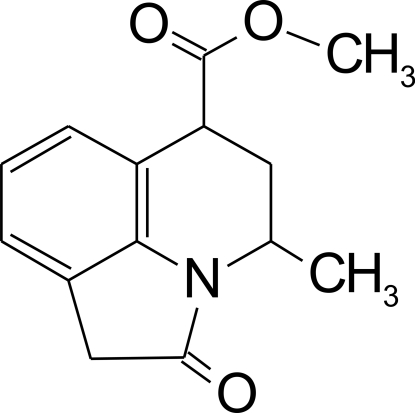


## Experimental

### 

#### Crystal data

C_14_H_15_NO_3_
*M* *_r_* = 245.27
Monoclinic, 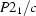

*a* = 8.309 (3) Å
*b* = 18.524 (5) Å
*c* = 8.730 (3) Å
β = 112.30 (3)°
*V* = 1243.2 (8) Å^3^
*Z* = 4
Mo *K*α radiation
μ = 0.09 mm^−1^
*T* = 295 K
0.20 × 0.20 × 0.20 mm

#### Data collection

Enraf–Nonius CAD-4 diffractometer
Absorption correction: none
2699 measured reflections
2445 independent reflections
1974 reflections with *I* > 2σ(*I*)
*R*_int_ = 0.030
1 standard reflections frequency: 60 min intensity decay: 2%

#### Refinement

*R*[*F* ^2^ > 2σ(*F* ^2^)] = 0.048
*wR*(*F* ^2^) = 0.174
*S* = 1.09
2445 reflections
165 parameters
H-atom parameters constrained
Δρ_max_ = 0.29 e Å^−3^
Δρ_min_ = −0.33 e Å^−3^

### 

Data collection: *CAD-4 EXPRESS* (Enraf–Nonius, 1994 ▶); cell refinement: *CAD-4 EXPRESS*; data reduction: *XCAD4* (Harms & Wocadlo, 1995 ▶); program(s) used to solve structure: *SHELXS97* (Sheldrick, 2008 ▶); program(s) used to refine structure: *SHELXL97* (Sheldrick, 2008 ▶); molecular graphics: *ORTEP-3* (Farrugia, 1997 ▶); software used to prepare material for publication: *WinGX* (Farrugia, 1999 ▶).

## Supplementary Material

Crystal structure: contains datablocks global, I. DOI: 10.1107/S1600536809029948/cv2589sup1.cif

Structure factors: contains datablocks I. DOI: 10.1107/S1600536809029948/cv2589Isup2.hkl

Additional supplementary materials:  crystallographic information; 3D view; checkCIF report

## Figures and Tables

**Table 1:** Hydrogen-bond geometry (Å, °)

*D*—H⋯*A*	*D*—H	H⋯*A*	*D*⋯*A*	*D*—H⋯*A*
C3—H3*A*⋯*Cg*^i^	0.97	2.55	3.514 (3)	174
C4—H4⋯O12^ii^	0.98	2.40	3.274 (3)	149
C11—H11*A*⋯O12^iii^	0.97	2.56	3.394 (3)	145

Symmetry codes: (i) 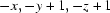; (ii) 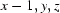; (iii) 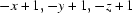. *Cg* is the centroid of the C5–C10 ring.

**Fig. 1.**
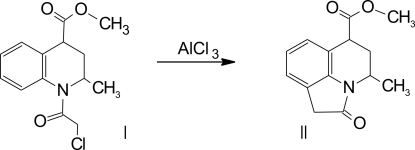
Synthesis of II.

**Fig. 2.**
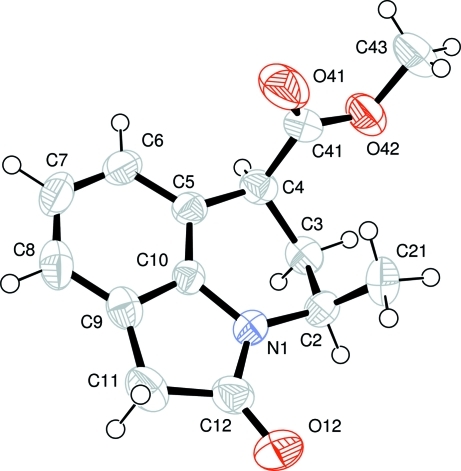
Molecular structure of the title compound II, showing the atom numbering scheme. Displacement ellipsoids are drawn at the 50% probability level. The H atoms are presented as a small spheres of arbitrary radius.

## supplementary crystallographic information

### Comment

Alkaloids have diverse and impotant physiological activity but it is impossible to fill alkaloids demand from natural source. This causes an attention to new synthetic methods and investigation of similar compounds. Methyl 2-oxo-1,2,5,6-tetrahydro-4H-pyrrolo[3,2,1-ij]quinoline-6-carboxylate (II) was prepared from the previously synthesized substituted 1,2,3,4-tetrahydroquinoline-4-carboxylic acid (I) - Fig. 1. The configuration of substituents cannot be resolved unambiguously by NMR. In accordance with the ^1^H NMR and GC-MS data starting I was pure *cis*-isomer (Zhuravleva *et al.*, 2009). Only one entry (Bond *et al.*, 1979) with the same heterothricycllic moiety was found in CSDB (ver. 5.30; Allen, 2002). All geometric parameters - the same bonds and angles are identical in s.u. intervals.

In the crystal, the C–H···π (centroid of C5-10) interactions (Table 1) link the molecules into centrosymmetric dimers. Weak intermolecular C–H···O hydrogen bonds (Table 1) link further these dimers into columns propagated in direction [100] .

### Experimental

To a stirred solution of *cis*-methyl 1-(chloroacetyl)-2-methyl-1,2,3,4-tetrahydroquinoline-4-carboxylate (3.6 mmol) in 1,2-dichlorobenzene (20 ml) aluminium chloride (36 mmol) was added dropwise at 378 K. The resulting mixture was stirred for 5 h at 378 K. To the cooled reaction mixture was added ice-water and adjusted to *pH* 10 with solution of sodium hydrocarbonate. The mixture was extracted with ethyl acetate, dried over anhydrous sodium sulfate and concentrated under reduced pressure. Recrystallization of the crude product from ethanol gave 0.73 g of colourless crystals. Yield 73%, mp 398-341 K.

IR, ν, cm^-1^: 1731 (CO), 1701 (NCO). MS, m/*z*: 245 (100) [*M*]+, 230 (40), 187 (7), 186 (84), 170 (66), 158 (91), 142 (46). ^1^H NMR, δ: 1.15 d (3*H*, CH_3_), 2.09-2.20 m (1*H*, 3-CH_2_), 2.30-2.40 m (1*H*, 3-CH), 3.50 s (1*H*, CH_2_), 3.55 s (1*H*, CH_2_), 3.65 s (3*H*, OCH_3_), 3.96-4.01 m (1*H*, 2-CH), 4.17-4.27 m (1*H*, 4-CH), 6.95 t (1*H*, 6-H), 7.13 pt (2*H*, 7-H, 5-H). Anal. calc. for C_14_H_15_NO_3_, %: C 69.21; H 6.37; N 5.53. Found, %: C 68.57; H 6.12; N 5.71.

Single crystals for *X*-ray analysis were obtained by slow evaporation of an methylene chloride - hexane (2: 3) solution. IR spectrum was recorded (in KBr) on Shimadzu FTIR-8400S. Mass spectrum was measured on Finnigan Trance DSQ spectrometer. ^1^H NMR spectrum was obtained in *DMSO*-*d*_6_ on Bruker AM 300 (300 MHz), using *TMS* as internal standard. Elemental composition was determined on Euro Vector EA-3000 elemental analyzer.

### Refinement

All H-atoms were geometrically positioned and refined using a riding model with d(C–H) = 0.93 Å, *U*_iso_(H) = 1.2*U*_eq_(C) for aromatic, d(C–H) = 0.97 Å, *U*_iso_(H) = 1.2*U*_eq_(C) for CH_2_, d(C–H) = 0.96 Å, *U*_iso_(H) = 1.5*U*_eq_(C) for CH_3_ atoms.

### Crystal data

**Table:**

C_14_H_15_NO_3_	*F*(000) = 520
*M**_r_* = 245.27	*D*_x_ = 1.310 Mg m^−^^3^
Monoclinic, *P*2_1_/*c*	Mo *K*α radiation, λ = 0.71073 Å
Hall symbol: -P 2ybc	Cell parameters from 25 reflections
*a* = 8.309 (3) Å	θ = 19.8–20.5°
*b* = 18.524 (5) Å	µ = 0.09 mm^−^^1^
*c* = 8.730 (3) Å	*T* = 295 K
β = 112.30 (3)°	Prism, yellow
*V* = 1243.2 (8) Å^3^	0.20 × 0.20 × 0.20 mm
*Z* = 4	

### Data collection

**Table:**

Enraf–Nonius CAD-4 diffractometer	*R*_int_ = 0.030
Radiation source: Fine-focus sealed tube	θ_max_ = 26.0°, θ_min_ = 2.2°
Graphite	*h* = −10→9
Nonprofiled ω scans	*k* = 0→22
2699 measured reflections	*l* = 0→10
2445 independent reflections	1 standard reflections every 60 min
1974 reflections with *I* > 2σ(*I*)	intensity decay: 2%

### Refinement

**Table:**

Refinement on *F*^2^	Primary atom site location: Direct
Least-squares matrix: Full	Secondary atom site location: Difmap
*R*[*F*^2^ > 2σ(*F*^2^)] = 0.048	Hydrogen site location: Geom
*wR*(*F*^2^) = 0.174	H-atom parameters constrained
*S* = 1.09	*w* = 1/[σ^2^(*F*_o_^2^) + (0.1046*P*)^2^ + 0.277*P*] where *P* = (*F*_o_^2^ + 2*F*_c_^2^)/3
2445 reflections	(Δ/σ)_max_ = 0.003
165 parameters	Δρ_max_ = 0.29 e Å^−^^3^
0 restraints	Δρ_min_ = −0.32 e Å^−^^3^

### Special details

**Table:**

Geometry. All s.u.'s (except the s.u. in the dihedral angle between two l.s. planes) are estimated using the full covariance matrix. The cell s.u.'s are taken into account individually in the estimation of s.u.'s in distances, angles and torsion angles; correlations between s.u.'s in cell parameters are only used when they are defined by crystal symmetry. An approximate (isotropic) treatment of cell s.u.'s is used for estimating s.u.'s involving l.s. planes.
Refinement. Refinement of *F*^2^ against ALL reflections. The weighted *R*-factor *wR* and goodness of fit *S* are based on *F*^2^, conventional *R*-factors *R* are based on *F*, with *F* set to zero for negative *F*^2^. The threshold expression of *F*^2^ > 2σ(*F*^2^) is used only for calculating *R*-factors(gt) *etc*. and is not relevant to the choice of reflections for refinement. *R*-factors based on *F*^2^ are statistically about twice as large as those based on *F*, and *R*-factors based on ALL data will be even larger.

### Fractional atomic coordinates and isotropic or equivalent isotropic displacement parameters (Å^2^)

**Table:**

	*x*	*y*	*z*	*U*_iso_*/*U*_eq_	
N1	0.1569 (2)	0.42646 (8)	0.3299 (2)	0.0427 (4)	
C2	0.1249 (3)	0.35798 (10)	0.3939 (2)	0.0465 (5)	
H2	0.2271	0.3470	0.4937	0.056*	
C21	0.1052 (3)	0.29783 (11)	0.2711 (3)	0.0580 (6)	
H21A	−0.0013	0.3043	0.1768	0.087*	
H21B	0.1027	0.2522	0.3223	0.087*	
H21C	0.2016	0.2988	0.2360	0.087*	
C3	−0.0291 (3)	0.36744 (11)	0.4463 (2)	0.0475 (5)	
H3A	0.0069	0.3977	0.5443	0.057*	
H3B	−0.0600	0.3206	0.4765	0.057*	
C4	−0.1924 (3)	0.40114 (11)	0.3133 (2)	0.0453 (5)	
H4	−0.2691	0.4158	0.3697	0.054*	
C41	−0.2951 (3)	0.34861 (11)	0.1782 (3)	0.0505 (5)	
O41	−0.3534 (3)	0.36109 (10)	0.0332 (2)	0.0836 (6)	
O42	−0.3192 (2)	0.28667 (8)	0.2428 (2)	0.0678 (5)	
C43	−0.4180 (4)	0.23173 (15)	0.1274 (4)	0.0925 (10)	
H43A	−0.5231	0.2525	0.0496	0.139*	
H43B	−0.4466	0.1935	0.1869	0.139*	
H43C	−0.3499	0.2128	0.0691	0.139*	
C5	−0.1448 (2)	0.46862 (10)	0.2453 (2)	0.0407 (4)	
C6	−0.2560 (3)	0.52609 (12)	0.1702 (3)	0.0554 (6)	
H6	−0.3722	0.5238	0.1578	0.067*	
C7	−0.1959 (3)	0.58653 (12)	0.1140 (3)	0.0630 (7)	
H7	−0.2727	0.6239	0.0642	0.076*	
C8	−0.0246 (3)	0.59218 (11)	0.1306 (2)	0.0557 (6)	
H8	0.0139	0.6327	0.0917	0.067*	
C9	0.0883 (3)	0.53697 (10)	0.2055 (2)	0.0443 (5)	
C10	0.0247 (2)	0.47699 (9)	0.2586 (2)	0.0362 (4)	
C11	0.2786 (3)	0.52466 (12)	0.2464 (3)	0.0558 (6)	
H11A	0.3485	0.5608	0.3233	0.067*	
H11B	0.3050	0.5256	0.1473	0.067*	
C12	0.3104 (3)	0.45028 (12)	0.3248 (3)	0.0521 (5)	
O12	0.4456 (2)	0.41618 (10)	0.3776 (3)	0.0769 (6)	

### Atomic displacement parameters (Å^2^)

**Table:**

	*U*^11^	*U*^22^	*U*^33^	*U*^12^	*U*^13^	*U*^23^
N1	0.0376 (8)	0.0402 (9)	0.0496 (9)	0.0039 (6)	0.0159 (7)	0.0012 (7)
C2	0.0446 (10)	0.0439 (11)	0.0427 (10)	0.0043 (8)	0.0071 (8)	0.0094 (8)
C21	0.0725 (15)	0.0358 (10)	0.0690 (14)	0.0055 (10)	0.0308 (12)	−0.0003 (9)
C3	0.0561 (12)	0.0499 (11)	0.0356 (9)	−0.0064 (9)	0.0164 (8)	0.0018 (8)
C4	0.0408 (10)	0.0489 (11)	0.0491 (11)	−0.0034 (8)	0.0203 (8)	−0.0069 (8)
C41	0.0396 (10)	0.0497 (12)	0.0563 (13)	−0.0070 (8)	0.0115 (9)	−0.0031 (9)
O41	0.0950 (14)	0.0728 (12)	0.0569 (11)	−0.0272 (10)	−0.0007 (9)	−0.0017 (9)
O42	0.0704 (11)	0.0501 (9)	0.0745 (11)	−0.0186 (8)	0.0183 (9)	−0.0025 (8)
C43	0.0841 (19)	0.0559 (15)	0.111 (2)	−0.0299 (14)	0.0071 (17)	−0.0089 (15)
C5	0.0384 (9)	0.0415 (10)	0.0374 (9)	0.0009 (7)	0.0090 (7)	−0.0070 (7)
C6	0.0436 (11)	0.0502 (12)	0.0577 (13)	0.0093 (9)	0.0026 (9)	−0.0108 (10)
C7	0.0725 (16)	0.0412 (11)	0.0519 (13)	0.0150 (10)	−0.0029 (11)	−0.0007 (9)
C8	0.0873 (17)	0.0346 (10)	0.0378 (10)	−0.0015 (10)	0.0154 (10)	0.0016 (8)
C9	0.0595 (12)	0.0382 (10)	0.0371 (9)	−0.0051 (8)	0.0203 (9)	−0.0064 (7)
C10	0.0413 (10)	0.0335 (9)	0.0320 (8)	0.0013 (7)	0.0119 (7)	−0.0033 (7)
C11	0.0624 (14)	0.0507 (12)	0.0648 (13)	−0.0134 (10)	0.0358 (11)	−0.0086 (10)
C12	0.0427 (11)	0.0525 (12)	0.0652 (13)	−0.0049 (9)	0.0251 (10)	−0.0103 (10)
O12	0.0426 (9)	0.0747 (12)	0.1144 (15)	0.0075 (8)	0.0308 (9)	−0.0048 (10)

### Geometric parameters (Å, °)

**Table:**

N1—C12	1.366 (3)	C43—H43A	0.9600
N1—C10	1.397 (2)	C43—H43B	0.9600
N1—C2	1.451 (2)	C43—H43C	0.9600
C2—C21	1.512 (3)	C5—C10	1.377 (3)
C2—C3	1.523 (3)	C5—C6	1.399 (3)
C2—H2	0.9800	C6—C7	1.389 (4)
C21—H21A	0.9600	C6—H6	0.9300
C21—H21B	0.9600	C7—C8	1.379 (4)
C21—H21C	0.9600	C7—H7	0.9300
C3—C4	1.544 (3)	C8—C9	1.374 (3)
C3—H3A	0.9700	C8—H8	0.9300
C3—H3B	0.9700	C9—C10	1.383 (3)
C4—C5	1.500 (3)	C9—C11	1.501 (3)
C4—C41	1.517 (3)	C11—C12	1.517 (3)
C4—H4	0.9800	C11—H11A	0.9700
C41—O41	1.193 (3)	C11—H11B	0.9700
C41—O42	1.327 (3)	C12—O12	1.217 (3)
O42—C43	1.449 (3)		
			
C12—N1—C10	110.78 (17)	O42—C43—H43B	109.5
C12—N1—C2	127.21 (17)	H43A—C43—H43B	109.5
C10—N1—C2	121.98 (16)	O42—C43—H43C	109.5
N1—C2—C21	110.93 (17)	H43A—C43—H43C	109.5
N1—C2—C3	108.22 (16)	H43B—C43—H43C	109.5
C21—C2—C3	114.86 (18)	C10—C5—C6	115.24 (19)
N1—C2—H2	107.5	C10—C5—C4	118.39 (16)
C21—C2—H2	107.5	C6—C5—C4	126.37 (19)
C3—C2—H2	107.5	C7—C6—C5	121.1 (2)
C2—C21—H21A	109.5	C7—C6—H6	119.4
C2—C21—H21B	109.5	C5—C6—H6	119.4
H21A—C21—H21B	109.5	C8—C7—C6	121.3 (2)
C2—C21—H21C	109.5	C8—C7—H7	119.4
H21A—C21—H21C	109.5	C6—C7—H7	119.4
H21B—C21—H21C	109.5	C9—C8—C7	119.0 (2)
C2—C3—C4	114.81 (16)	C9—C8—H8	120.5
C2—C3—H3A	108.6	C7—C8—H8	120.5
C4—C3—H3A	108.6	C8—C9—C10	118.6 (2)
C2—C3—H3B	108.6	C8—C9—C11	133.9 (2)
C4—C3—H3B	108.6	C10—C9—C11	107.45 (17)
H3A—C3—H3B	107.5	C5—C10—C9	124.77 (17)
C5—C4—C41	112.46 (17)	C5—C10—N1	124.60 (17)
C5—C4—C3	110.24 (15)	C9—C10—N1	110.62 (17)
C41—C4—C3	113.57 (17)	C9—C11—C12	103.46 (16)
C5—C4—H4	106.7	C9—C11—H11A	111.1
C41—C4—H4	106.7	C12—C11—H11A	111.1
C3—C4—H4	106.7	C9—C11—H11B	111.1
O41—C41—O42	123.5 (2)	C12—C11—H11B	111.1
O41—C41—C4	125.6 (2)	H11A—C11—H11B	109.0
O42—C41—C4	110.80 (19)	O12—C12—N1	124.0 (2)
C41—O42—C43	116.7 (2)	O12—C12—C11	128.3 (2)
O42—C43—H43A	109.5	N1—C12—C11	107.66 (18)
			
C12—N1—C2—C21	−78.4 (3)	C7—C8—C9—C10	1.3 (3)
C10—N1—C2—C21	99.7 (2)	C7—C8—C9—C11	−179.1 (2)
C12—N1—C2—C3	154.76 (19)	C6—C5—C10—C9	0.7 (3)
C10—N1—C2—C3	−27.2 (2)	C4—C5—C10—C9	−178.29 (17)
N1—C2—C3—C4	51.5 (2)	C6—C5—C10—N1	179.68 (17)
C21—C2—C3—C4	−73.1 (2)	C4—C5—C10—N1	0.7 (3)
C2—C3—C4—C5	−50.2 (2)	C8—C9—C10—C5	−1.4 (3)
C2—C3—C4—C41	77.1 (2)	C11—C9—C10—C5	178.81 (17)
C5—C4—C41—O41	−9.3 (3)	C8—C9—C10—N1	179.47 (16)
C3—C4—C41—O41	−135.4 (3)	C11—C9—C10—N1	−0.3 (2)
C5—C4—C41—O42	173.46 (17)	C12—N1—C10—C5	−179.89 (17)
C3—C4—C41—O42	47.4 (2)	C2—N1—C10—C5	1.8 (3)
O41—C41—O42—C43	1.8 (4)	C12—N1—C10—C9	−0.8 (2)
C4—C41—O42—C43	179.1 (2)	C2—N1—C10—C9	−179.16 (16)
C41—C4—C5—C10	−105.06 (19)	C8—C9—C11—C12	−178.6 (2)
C3—C4—C5—C10	22.8 (2)	C10—C9—C11—C12	1.1 (2)
C41—C4—C5—C6	76.1 (2)	C10—N1—C12—O12	−179.4 (2)
C3—C4—C5—C6	−156.10 (19)	C2—N1—C12—O12	−1.1 (4)
C10—C5—C6—C7	0.1 (3)	C10—N1—C12—C11	1.5 (2)
C4—C5—C6—C7	179.04 (19)	C2—N1—C12—C11	179.75 (18)
C5—C6—C7—C8	−0.2 (3)	C9—C11—C12—O12	179.4 (2)
C6—C7—C8—C9	−0.5 (3)	C9—C11—C12—N1	−1.6 (2)

### Hydrogen-bond geometry (Å, °)

**Table:**

*D*—H···*A*	*D*—H	H···*A*	*D*···*A*	*D*—H···*A*
C3—H3A···Cg^i^	0.97	2.55	3.514 (3)	174
C4—H4···O12^ii^	0.98	2.40	3.274 (3)	149
C11—H11A···O12^iii^	0.97	2.56	3.394 (3)	145

Symmetry codes: (i) −*x*, −*y*+1, −*z*+1; (ii) *x*−1, *y*, *z*; (iii) −*x*+1, −*y*+1, −*z*+1.
